# The vestigial lung of the coelacanth and its implications for understanding pulmonary diversity among vertebrates: new perspectives and open questions

**DOI:** 10.1098/rsos.171518

**Published:** 2017-11-22

**Authors:** Markus Lambertz

**Affiliations:** 1 Institut für Zoologie, Rheinische Friedrich-Wilhelms-Universität Bonn, Poppelsdorfer Schloss, 53115 Bonn, Germany; 2 Sektion Herpetologie, Zoologisches Forschungsmuseum Alexander Koenig, Adenauerallee 160, 53113 Bonn, Germany

The coelacanth, *Latimeria chalumnae* Smith, 1939 [1] (Sarcopterygii: Actinistia), together with the closely related *L. menadoensis* Pouyaud *et al*., 1999 [2], remains the only living representative of one of the most basally-branching primary radiations of lobe-finned fishes (Sarcopterygii). Even though extant species cannot be considered ‘primitive’ due to the inherent logic of phylogenetic theory, the coelacanth nonetheless is invaluable for understanding evolutionary transformations in basal sarcopterygians as it can help in the determination of character polarity. The appearance of one novelty during early vertebrate evolution that had major implications for the success of a huge number of species is the origin of lungs. The conventional interpretation is that lungs evolved in basal bony fishes (Osteichthyes or Osteognathostomata), were maintained in the lobe-finned fishes, and eventually were transformed into a swimbladder among the ray-finned fishes (Actinopterygii) (e.g. [3]). However, the currently available data do not rule out separate origins of lungs and swimbladders from a common ‘respiratory pharynx’, even though this would require a slightly less parsimonious course of evolution [4,5]. The coelacanth is a key species in addressing this question and for this reason the data recently provided by Cupello and colleagues [6] are a very welcome addition to the discussion. Here, I would like to add a few points pertinent to lung evolution that appear to be a consequence of these exciting data.

One of the most interesting aspects of the coelacanth is that it apparently exhibits an unpaired structure of putative homology with lungs [6–8]. In the Polypteriformes (bichir and reed fish), the lungs are paired [5,9,10], as are those of the lungfishes (Dipnoi) [11], except the Australian lungfish, *Neoceratodus forsteri* (Krefft, 1870) [12], in which only the right lung persists [11,13]. The *Anlage* of a left lung, however, is formed during early ontogeny also in this species, but rapidly degenerates during progressed development [13]. Similarly, it is well known that most species of snakes possess only a functional right lung [14] and several other serpentiform squamates also exhibit a reduction of one of their plesiomorphically paired lungs (e.g. [15,16]). Even more than in *N. forsteri*, however, also in these species, the *Anlagen* of both lungs still are formed embryologically. If one of the lungs persists in a vestigial form in the adult, it presents an arrested early developmental stage [17]. As Cupello and colleagues pointed out [6,8], the developmental stages of *L. chalumnae* presently available are rather far advanced and do not cover the critical early ontogenetic period. For this reason it is still not known how the unpaired condition of the vestigial lung in the coelacanth actually is to be interpreted. Is the unpaired condition the result of a direct development or does the coelacanth also exhibit a secondary reduction of one of its lungs? It has been speculated that the dual presence of an esophagial diverticulum (herein called the vestigial lung) and a fatty organ in coelacanths could indicate a paired lung homologue, one of which became fat-filled whereas the other one degenerated to (or persisted as) the small diverticulum [5]. The histological data available [6] appear to support such a hypothesis by reviving the earlier interpretation that also the apomorphic fatty organ is derived from the pulmonary complex [18]. The accumulation of fat in the wall of the vestigial lung of the coelacanth [6, fig. 2d] is not known for any other vertebrate lung and may indicate an apomorphic developmental trait for the pulmonary tissue in coelacanths that is also seen in the vestigial lung (as well as numerous other tissues [19]). The ‘main lung’ consequently could be represented by the fatty organ in which fat accumulation dominated the developmental process, resulting in a singular ‘solid’ organ with a buoyancy (and energy storage?) function. Such a scenario also would be in agreement with the palaeontological data on the pulmonary complex for early actinistians [20] and the hypothesis that a fatty organ became the dominant buoyancy regulator once the habitat was shifted towards deeper water depths [6,8]. Only embryological data on earlier developmental stages of the coelacanth, however, reliably can clarify the fate of the derivatives of the posterior pharynx including the origin of the unique fatty organ. This fatty organ extends around the vestigial lung [6,8,18], but so far it remains unresolved whether it develops independently from the posterior pharynx or even from the vestigial lung. Determining the actual condition of the pulmonary complex in the coelacanth (unpaired versus paired) is critical for an appropriate character coding of this complex in phylogenetic analyses. If the lung of the coelacanth and related extinct taxa is actually primarily unpaired and the fatty organ is independent of the pulmonary complex (or derived from the vestigial lung and not the pharynx), what does this mean for our interpretation of the paired lungs of the polypteriforms, lungfish and tetrapods? Are they really homologous and were they maintained from a common ancestor that had two lungs, one of which became completely reduced in the coelacanth? Or would this support the respiratory pharynx hypothesis, in which independent ‘natural experiments’ resulted in the maintenance of different protrusions of the posterior pharynx from a condition in which multiple of such protrusions were present, both on the ventral and dorsal side? The conspicuous accumulation of fat in the vestigial lung together with the presence of a fatty organ, both of which based on the currently available anatomical descriptions [6,8,18] nonetheless are potential derivatives of the ventral posterior pharynx, however, would be consistent with the paired *Anlage* of the remaining vertebrate lungs. Under such a scenario it would be expected that the fatty organ develops through a stage reminiscent of the vestigial lung, but that general growth and the deposition of fat continues and increases, and that this fatty organ eventually ‘encapsulates’ the vestigial lung.

Another interesting aspect concerning a major evolutionary question about vertebrate lungs that directly derives from the work of Cupello and colleagues is about the origin of intrapulmonary complexity. The revision of lung development and anatomy in all principal radiations of fully terrestrial vertebrates (Amniota) revealed that a common branched pulmonary *Bauplan* is shared by all of them and that the sac-like lungs of the tuatara, most lizards, and snakes (Lepidosauria) represent a secondary simplified condition of a plesiomorphically more complex one, which is still traceable embryologically [17]. Extant amphibians lack a corresponding developmental pattern and the lungs of adults do not present an internal structurization of their lungs comparable to that of the single-chambered ones of lepidosaurs. Lungfish, on the other hand, do show an internal surface elaboration that at least superficially can be compared to the ‘niches’ of many lizard lungs. These niches in lizard lungs, in turn, have been shown to be the direct adult representation of the embryonic intrapulmonary branching events [17]. The discovery of internal surface enlargements in the vestigial lung of the coelacanth [6] now raises the question as to which (or if any) of these structural elements of the sarcopterygian lungs can be homologized? Did intrapulmonary branching even predate the rise of amniotes and must in fact even be placed down to the root of the sarcopterygian tree? How should the amphibians be interpreted in such a scenario?

In conclusion, the recent data on the vestigial lung of *L. chalumnae* presented by Cupello and colleagues are invaluable contributions to our knowledge on the structure and evolution of vertebrate lungs. Their particular relevance lies in the fact that these studies now allow us to express an entire suite of further research questions that need to be addressed in the future. The origin of lungs is one of the most fundamental problems of vertebrate evolution as it was a prerequisite for the conquest of dry land and eventually also paved the way to our own rise, both on the basic physiological level (e.g. metabolic scope) as well as on the cultural one (vocal communication). The studies on the coelacanth, which is a key taxon for addressing all of these questions on early lung evolution, furthermore highlight the crucial importance of anatomical research that is based on museum specimens. It is hoped that if additional material of this elusive species becomes available in the future, the respective curators will permit their full utilization, not only accommodate their storage. One of the functions of natural history repositories undoubtedly is the archiving and documentation of biodiversity, but they also present priceless sources for comparative anatomical and thereby evolutionary morphological research. It should be noted that the ‘internal information’ contained in such voucher specimens constitutes a non-negligible dimension of their overall value, and that careful dissections may not only be viewed as a destructive process, but sometimes rather as necessary means to unveil these otherwise inaccessible but crucial data. The advances in digital approaches to study morphology over the past years have offered access to parts of these data in a non-destructive fashion, but even now, almost 200 years after the first introduction of the name [21], histology for instance remains invaluable in understanding animal form, function and ultimately also evolution.

## Data accessibility

This article has no additional data.

## References

[1] Smith JLB 1939 A living fish of Mesozoic type. Nature 143, 455–456. (doi:10.1038/143455a0)

[2] Pouyaud L, Wirjoatmodjo S, Rachmatika I, Tjakrawidjaja A, Hadiaty R, Hadie W 1999 Une nouvelle espèce de coelacanthe. Preuves génétiques et morphologiques. C. R. Acad. Sci. Paris Sciences de la vie/Life Sciences 322, 261–267. 10216801 10.1016/s0764-4469(99)80061-4

[3] Longo S, Riccio M, McCune AR 2013 Homology of lungs and gas bladders: insights from arterial vasculature. J. Morphol. 274, 687–703. (doi:10.1002/jmor.20128) 23378277 10.1002/jmor.20128

[4] Perry SF 2007 Swimbladder-lung homology in basal osteichthyes revisited. In Fish respiration and environment (eds Fernandes MN, Rantin FT, Glass ML, Kapoor BG), pp. 41–54. Enfield, NH: Science Publishers.

[5] Lambertz M, Perry SF 2015 ‘2016’ The lung-swimbladder issue: a simple case of homology – or not? In Phylogeny, anatomy and physiology of ancient fishes (eds Zaccone G, Dabrowski K, Hedrick MS, Fernandes JMO, Icardo JM), pp. 201–211. Boca Raton, FL: CRC Press.

[6] Cupello C, Meunier FJ, Herbin M, Clément G, Brito PM 2017 Lung anatomy and histology of the extant coelacanth shed light on the loss of air-breathing during deep-water adaptation in actinistians. R. Soc. open sci. 4, 161030 (doi:10.1098/rsos.161030) 28405393 10.1098/rsos.161030 PMC5383850

[7] Lambertz M 2015 Die Lunge des Quastenflossers *Latimeria chalumnae*. Naturwiss. Rundsch. 68, 626–628.

[8] Cupello C, Brito PM, Herbin M, Meunier FJ, Janvier P, Dutel H, Clément G 2015 Allometric growth in the extant coelacanth lung during ontogenetic development. Nat. Commun. 6, 8222 (doi:10.1038/ncomms9222) 26372119 10.1038/ncomms9222 PMC4647851

[9] Lechleuthner A, Schumacher U, Negele RD, Welsch U 1989 Lungs of *Polypterus* and *Erpetoichthys*. J. Morphol. 201, 161–178. (doi:10.1002/jmor.1052010206) 10.1002/jmor.1052010206 29865640

[10] Icardo JM, Colvee E, Kuciel M, Lauriano ER, Zaccone G 2017 The lungs of *Polypterus senegalus* and *Erpetoichthys calabaricus*: insights into the structure and functional distribution of the pulmonary epithelial cells. J. Morphol. 278, 1321–1332. (doi:10.1002/jmor.20715) 28568283 10.1002/jmor.20715

[11] Spencer B 1898 Der Bau der Lungen von *Ceratodus* und *Protopterus*. In Zoologische Forschungsreisen in Australien und dem malayischen Archipel. Erster Band: Ceratodus. II. Lieferung (ed. Semon R), *Denkschr. medic.-naturwiss. Ges. Jena* **4**, 51–58 + Pl. IX–X.

[12] Krefft G 1870 To the Editor of the Herald. *The Sydney Morning Herald* 9878, 5.

[13] Neumayer L 1904 Die Entwickelung des Darmkanales, von Lunge, Leber, Milz und Pankreas bei *Ceratodus Forsteri*. In Zoologische Forschungsreisen in Australien und dem malayischen Archipel. Erster Band: Ceratodus. IV. Lieferung (ed. Semon R), *Denkschr. medic.-naturwiss. Ges. Jena* **4**, 377–422 + Pl. XXXVI

[14] Wallach V 1998 The lungs of snakes. In Biology of the Reptilia, vol. 19, Morphology G, visceral organs (eds Gans C, Gaunt AS), pp. 93–295. Ithaca, NY: Society for the Study of Amphibians and Reptiles.

[15] Milani A 1894 Beiträge zur Kenntniss der Reptilienlunge. I. Lacertilia. Zool. Jb. Abt. Anat. Ontog. Tiere 7, 545–592 + Pls. 30–32.

[16] Lambertz M, Arenz N, Grommes K In press Variability in pulmonary reduction and asymmetry in a serpentiform lizard: the sheltopusik, *Pseudopus apodus* (Pallas, 1775). Vert. Zool.

[17] Lambertz M, Grommes K, Kohlsdorf T, Perry SF 2015 Lungs of the first amniotes: why simple if they can be complex? Biol. Lett. 11, 20140848 (doi:10.1098/rsbl.2014.0848) 25568154 10.1098/rsbl.2014.0848 PMC4321151

[18] Millot J, Anthony T, Robineau D. 1978 Anatomie de Latimeria chalumnae, Tome III. Paris, France: CNRS.

[19] Millot J 1954 *Le troisième cœlacanthe – Historique, éléments d'ecologie, morphologie externe, documents divers*. *Le naturaliste Malgache*, Premier supplément, 26 p. + L Pls.

[20] Brito PM, Meunier FJ, Clément G, Geffard-Kuriyama D 2010 The histological structure of the calcified lung of the fossil coelacanth *Axelrodichthys araripensis* (*Actinistia: Mawsoniidae*). Palaeontology 53, 1281–1290. (doi:10.1111/j.1475-4983.2010.01015.x)

[21] Mayer C 1819 Ueber Histologie und eine neue Eintheilung der Gewebe des menschlichen Körpers. Bonn, Germany: Adolph Marcus.

